# Variable telomere length across post-mortem human brain regions and specific reduction in the hippocampus of major depressive disorder

**DOI:** 10.1038/tp.2015.134

**Published:** 2015-09-15

**Authors:** F Mamdani, B Rollins, L Morgan, R M Myers, J D Barchas, A F Schatzberg, S J Watson, H Akil, S G Potkin, W E Bunney, M P Vawter, P A Sequeira

**Affiliations:** 1 Functional Genomics Laboratory, Department of Psychiatry and Human Behavior, University of California, Irvine, Irvine, CA, USA; 2 Hudson Alpha Institute for Biotechnology, Huntsville, AL, USA; 3 Department of Psychiatry, Weill Cornell Medical College, New York, NY, USA; 4 Department of Psychiatry & Behavioral Sciences, Stanford University, Palo Alto, CA, USA; 5 Molecular and Behavioral Neurosciences Institute, Department of Psychiatry, University of Michigan, Ann Arbor, MI, USA; 6 Department of Psychiatry and Human Behavior, University of California, Irvine, Irvine, CA, USA

## Abstract

Stress can be a predisposing factor to psychiatric disorders and has been associated with decreased neurogenesis and reduced hippocampal volume especially in depression. Similarly, in white blood cells chronic psychological stress has been associated with telomere shortening and with mood disorders and schizophrenia (SZ). However, in previous post-mortem brain studies from occipital cortex and cerebellum, no difference in telomere length was observed in depression. We hypothesized that in psychiatric disorders, stress-driven accelerated cellular aging can be observed in brain regions particularly sensitive to stress. Telomere length was measured by quantitative-PCR in five brain regions (dorsolateral prefrontal cortex, hippocampus (HIPP), amygdala, nucleus accumbens and substantia nigra (SN)) in major depressive disorder (MDD), bipolar disorder, SZ and normal control subjects (*N*=40, 10 subjects per group). We observed significant differences in telomere length across brain regions suggesting variable levels of cell aging, with SN and HIPP having the longest telomeres and the dorsolateral prefrontal cortex the shortest. A significant decrease (*P*<0.02) in telomere length was observed specifically in the HIPP of MDD subjects even after controlling for age. In the HIPP of MDD subjects, several genes involved in neuroprotection and in stress response (FKBP5, CRH) showed altered levels of mRNA. Our results suggest the presence of hippocampal stress-mediated accelerated cellular aging in depression. Further studies are needed to investigate the cellular specificity of these findings.

## Introduction

Telomeres are special sequences of DNA located at the end of chromosomes that preserve DNA integrity. Telomeres naturally shrink with age and cell division eventually leading to cell death.^1^ Shortening of telomeres can occur due to exposure to stress especially to oxidative stress, since the telomeric sequence is susceptible to oxidative damage^2^ and that telomeres undergo double stranded DNA breaks in the absence of replication due to oxidative stress.^3^ This has led to the proposition that telomere shortening can occur in cells not undergoing active mitosis, such as adult neurons, when exposed to chronic stress.^4^ Studies have shown that stress and increased cortisol levels can lead to the shortening of telomeres in lymphocytes^5^ and there is evidence for decreased telomere length in leukocytes of mood disorders and schizophrenia (SZ) patients.^6, 7, 8^ Most studies investigating telomere length have been performed using blood leukocytes and have shown shorter telomeres associated with major depressive disorder (MDD).^4^ Thus far, in brain tissue, no decrease in telomere length has been observed in studies quantifying telomere length in the cerebellum^9^ and the occipital cortex^10^ of subjects with psychiatric disorders. However, a recent study found shorter telomeres in oligodendrocytes of MDDs compared with controls in white matter from BA10 and the temporal lobe.^11^

Telomere length is highly dependent on the telomerase enzyme activity. This enzyme is responsible for adding the telomere repeat sequence (TTAGGG) at the 3′ end of chromosomes. Telomerase is composed of a catalytic protein component with reverse transcriptase properties (hTERT) and an RNA component (hTERC). In somatic cells, hTERT is not expressed but hTERC has been shown to be associated with telomere length. Defects in hTERC lead to shorter telomeres. Genetic variation in hTERC and has also been shown to be associated with telomere length.^12, 13, 14^

Telomere length is also regulated by the shelterin complex, a group of six proteins forming a cap to protect telomeres from DNA damage.^15^ The shelterin complex consists of TERF1 (telomeric-repeat-binding factor 1), TERF2, TIN2 (TRF1-interacting protein 2), TERF2IP (TERF2-interacting protein), POT1 (protection of telomeres 1) and, the POT1- and TIN2-organizing protein TPP1 (also known as ACD).^15^ Three of these proteins (TERF1, TERF2 and POT1) recognize the telomeric repeat TTAGGG while the other three proteins (TIN2, TPP1 and RAP1) serve as connectors for the first three to form the shelterin complex.^15^

In peripheral tissues, such as blood cells, telomere length decreases with age because of successive cell divisions.^16^ However, in brain tissue which is mainly composed of neurons and glia, two cell types that do not divide constantly, no correlation between age and telomere shortening has been observed in gray matter.^9, 17^

In this study, we investigated telomere length across five brain regions, amygdala (AMY), dorsolateral prefrontal cortex (DLPFC), hippocampus (HIPP), nucleus accumbens (NAC) and substantia nigra (SN) in subjects with neuropsychiatric disorders (MDD, bipolar disorder (BD) and SZ), and in control subjects. We hypothesized that there would be a decrease in telomere length in psychiatric disorders, possibly as a result of stress associated with psychiatric diagnoses. Furthermore, we explored the relationship between telomere length and expression levels of genes involved in telomere maintenance and in stress response.

## Materials and methods

### Study sample

The subjects used for this study consisted of post-mortem human brains from MDD (*N*=10), BD (*N*=10), SZ (*N*=10) and psychiatrically normal controls (*N*=10) (Table 1). The brains were collected by the University of California, Irvine Brain Bank (UCIBB) in accordance with the university's Institutional Review Board after obtaining consent from next of kin. These brains have been characterized using the UCIBB psychological autopsy protocol, which is largely based on procedures validated by Kelly and Mann^18^ and has been extensively used by our group in past years.^19, 20, 21, 22, 23, 24, 25^ The psychological autopsy protocol enables the accurate post-mortem diagnosis of subjects based on multiple sources of information, with diagnosis being agreed on through consensus between independent clinical psychologists and a psychiatrist. For each subject, five brain regions were dissected—DLPFC, HIPP, AMY, NAC and SN. These regions were chosen due to their involvement in neuropsychiatric disorders. Dissection of the areas-of-interest was done on dry ice using neuroanatomical landmarks and being blinded to diagnostic group.

### Telomere length assay

DNA was extracted specifically for this project, to avoid batch effects, from 30 mg of fresh-frozen tissue using the DNeasy Blood and Tissue DNA extraction kit (Qiagen, Valencia, CA, USA). Briefly, tissue was diced into very small pieces and incubated overnight at 56 °C in lysis buffer containing Proteinase K and subsequently column purified to obtain DNA. DNA concentration was measured using a spectrophotometer and standardized working solutions of 10 ng μl^−1^ were aliquoted into 96-well plates for ease of use during PCR.

Telomere length was determined using quantitative real-time PCR and a protocol based on that proposed by Cawthon.^26^ Specifically, two genomic assays were carried out, one for the human albumin gene (*ALB*), which is a single copy gene, and the other assay with primers specific to the repetitive telomeric (*TEL*) sequence. A ratio of the relative quantities (TEL/ALB) was then used as a quantitative measure of telomere length.

The primers used to amplify the single copy gene were: *ALB*F (5′-CTGTCATCTCTTGTGGGCTGT-3′) and *ALB*R (5′-GGCATGACAGGTTTTGCAATA-3′) and those for the telomeric sequence were: *TEL1*b (5′-CGGTTTGTTTGGGTTTGGGTTTGGGTTTGGGTTTGGGTT-3′) and *TEL2*b (5′-GGCTTGCCTTACCCTTACCCTTACCCTTACCCTTACCCT-3′). The quantitative real-time PCRs were performed on an Applied Biosystems 7900HT real-time machine (Waltham, MA, USA) in 12.5 μl reactions. The ALB quantitative PCR was run using the following cycling parameters; 95 °C for 10 min, followed by 40 cycles of denaturation at 95 °C for 15 s and annealing/extention at 60 °C for 1 min. For TEL, the quantitative PCR was run using the following cycling parameters; 95 °C for 10 min, followed by 40 cycles of denaturation at 95 °C for 15 s and annealing/extention at 56 °C for 1 min. Within each plate, a standard curve with seven data points, ranging from 40 to 0.625 ng of pooled DNA, was run and used for the calculation of reaction efficiencies for comparisons between plates. Each sample was run in triplicate and an average of the cycle thresholds was used to calculate the telomere/single copy gene (T/S) ratios as described in Cawthon.^26^ This method has the advantage of requiring small amounts of DNA and provides telomere length estimates that are highly correlated with mean TRF lengths as determined by Southern blot analysis.^26^

### RNA quantification

RNA was extracted from 80 to 100 mg of tissue using Trizol reagent (Life Technologies, Carlsbad, CA, USA). Tissue was homogenized in Trizol using an Omni-Prep multisample homogenizer (Omni International, Kennesaw, GA, USA) and extracted as per the manufacturer's protocol. RNA concentration was determined using Qubit (Life Technologies) and RNA integrity was assessed using an Agilent Bioanalyzer (Agilent Technologies, Santa Clara, CA, USA).

RNA (5 μl at a concentration of 20 ng μl^−1^) was directly quantified using the NanoString nCounter analysis system (NanoString, Seattle, WA, USA). The NanoString platform allows to directly (no reverse transcription needed) and digitally quantify the number of mRNA molecules present in a sample in a highly multiplexed way (up to 800 targets) in a single reaction using color molecular barcodes. Because no complementary DNA is synthesized, RNA quantification using NanoString is less sensitive to RNA degradation and factors affecting RNA quality in paraffin-embedded samples or post-mortem samples like fixing procedures, post-mortem interval (PMI) and pH.^27, 28^ Samples were processed at the Genomics High-Throughput Facility of the University of California, Irvine. NanoString probes (Supplementary Table 1) were custom designed to include the following 11 telomere-associated genes: TERT, TERC, NHP2, TERF1, TINF2, TERF2, POT1, TERF2IP, nucleolar protein family A member 1 (GAR1/NOLA1), NHP2/NOLA2 and NOP10/NOLA3. We also included the following 10 stress and neuroprotection-related genes: FKBP5 (FK506-binding protein 5), CRH (corticotropin-releasing hormone), HSPA2 (heatshock 70 kDa protein 2), NR3C1 (nuclear receptor subfamily 3, group C, member 1), NR3C2, PPARA (peroxisome proliferator-activated receptor, alpha), PPARG, PPARD, RXRA (retinoid X receptor, alpha), NPY (neuropeptide Y) and GPR37 (G-protein coupled receptor 37).

### Statistical analysis

Nanostring data were processed using nSolver analysis software version 1.1 from NanoString for quality control and with Partek Genomic Suite 6.6 (Partek, St-Louis, MO, USA) for normalization and statistical analysis using normalization parameters suggested by NanoString. Analysis of variance was used to investigate the effect of demographic variables, pH and PMI on telomere length and gene expression levels. Telomere length shortening between the different diagnoses and controls were also investigated by analysis of variance with and without covariates to correct for possible confounders.

Power analysis^29, 30, 31^ was carried out using Partek Genomics Suite 6.6 with the following parameters; control vs MDD comparison, an effect size range of 1.25 to 3.0 (by step 0.25) and a sample size ranging from 8 to 92 (by step 10). This resulted in the determination that an 80% power at a significance of 0.01 could be obtained with an *N*=6.

## Results

**Figure 1:**
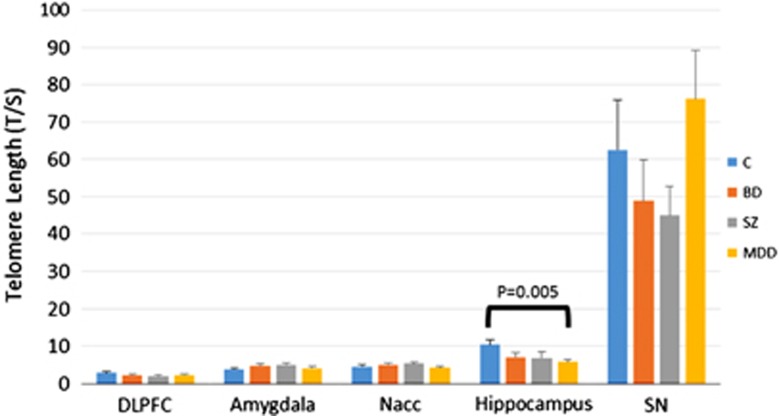
Distribution of telomere length across five brain regions in controls (C), bipolar disorder (BD), major depressive disorder (MDD) and Schizophrenia (SZ). Significant differences in average telomere length are observed across brain regions. The DLPFC has the shortest average telomere length (2.26) and the SN has the highest (58.14). In the hippocampus, we observed significantly shorter telomeres in MDD subjects compared with controls (*P*=0.0048, FC=−1.81). DLPFC, dorsolateral prefrontal cortex; FC, fold change; SN, substantia nigra.

Demographic variables as well as PMI and pH were not significantly different between groups (Table 1). Overall, telomere length, measured as T/S ratios, was not significantly affected by gender, PMI or pH. However, a small pH effect was found in the SN (Supplementary Table 2). Slightly negative correlations between age and telomere length were seen in each brain region (Supplementary Figure 1) but these correlations were not significant as previously reported.^9, 17, 32^ Brain regions showed highly significant difference in telomere length (*P*<0.001), with SN having the longest telomeres (average length=58.14) and DLPFC the shortest (average length=2.26) (Figure 1). No significant difference between controls and neuropsychiatric disorders for telomere length were observed in the DLPFC, AMY, NAC or SN (Table 2). A marginal increase in telomere length was observed in the AMY of SZ subjects (*P*=0.05, fold change=1.44). However, as expected given the changes observed in leukocytes, in the HIPP of MDDs significantly shorter telomeres were observed (*P*=0.0048, fold change=−1.81, Table 2 and Supplementary Figure 2) and this decrease was significant after correcting for possible confounding variable (gender, age, pH and PMI (*P*=0.02, fold change=−1.87, Table 2 and Supplementary Figure 2). The observed *P*-value (*P*=0.0048) passes Bonferroni correction for one tail *t*-test for the number of comparisons (0.1/15=0.006). Gender effects were particularly investigated when we compared controls with MDD, the interaction diagnosis × gender was not significant.

### Gene expression

**Figure 2:**
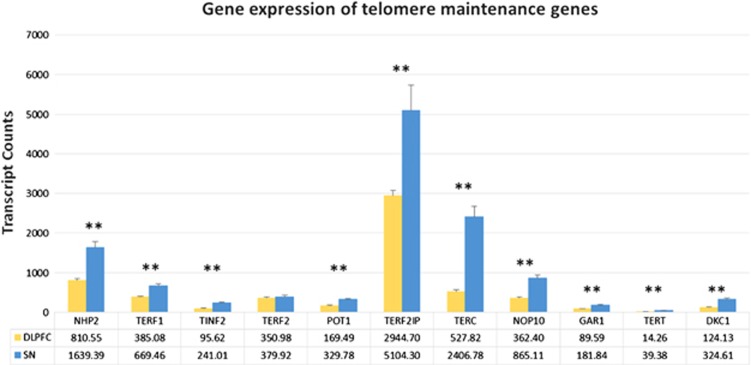
Gene expression based on NanoString transcript counts for telomere maintenance genes in the DLPFC and SN in controls. Expression levels of telomere maintenance genes have lower expression in the DLPFC compared with the SN, which could be a reason for the increased telomere length seen in the SN, (**P-value⩽0.005). DLPFC, dorsolateral prefrontal cortex; SN, substantia nigra.

Gene expression was investigated to explore the potential effects of telomere maintenance genes and stress-associated genes on telomere length in particular with regards to observed regional differences and those between MDDs and controls. The DLPFC and SN were the brain regions with the shortest and longest telomeres, respectively. A comparison of the RNA levels of telomere maintenance genes between these regions showed lower gene expression of several key telomere maintenance genes in the DLPFC compared with the SN (Figure 2). TERT was not detected at reliable levels which is normal for adult somatic tissue and TERF2 was not significantly different between the DLPFC and SN but other members of the telomerase complex (TERC, GAR1, DKC1, NHP2 and NOP10) and shelterin complex (TPP1, TERF1, TERF2IP, TINF2 and POT1) were expressed at higher levels in the SN when compared with the DLPFC.

**Figure 3:**
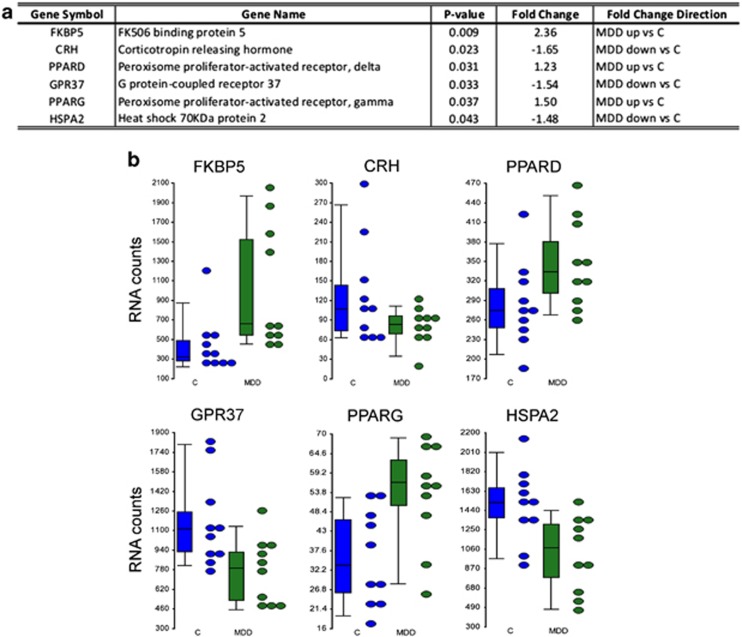
Genes found to be significantly different between MDD and controls in the hippocampus. (**a**) Table of *P*-values and fold changes for the significant genes. (**b**) Dot plots of genes which are involved in stress response and neuroprotection alluding to a state of heightened stress in MDDs requiring the preservation of neurons. MDD, major depressive disorder.

In the HIPP, a significant effect of diagnosis was observed for several key stress and stress response genes when comparing MDDs with controls. This effect remained significant after controlling for telomere length, age, gender, suicide, pH and PMI. The hypothesis for this comparison was that the shorter telomere length observed in the HIPP of MDDs was possibly due to gene expression alterations in telomere maintenance genes or in stress-related genes. This was found to be true especially for several stress response and neuroprotection genes, specifically FKBP5, CRH, HSPA2, PPARD, PPARG and GPR37 (Supplementary Table 1) that were significantly differentially expressed between MDDs and controls (Figure 3).

## Discussion

We quantitatively surveyed telomere length in a cohort of psychiatric patients (MDD, BD and SZ) and normal controls and observed a significant 1.8-fold telomere length reduction in the HIPP of MDDs and also noted variable telomere length across the brain regions investigated. This is to our knowledge the first evidence linking telomere length reductions in the human HIPP to major depression. Interestingly, in a recent article Wei *et al.*^33^ also report shorter telomeres in the HIPP of a rodent genetic model of depression. Previous clinical studies have consistently observed shorter telomeres in leukocytes of mood disorder patients^8^ and also in SZ.^6^ However, studies carried out thus far in human brain tissue have not provided significant findings and have been conducted in brain regions which may be less involved in mental disorders, such as the occipital cortex^10^ and cerebellum.^9^ The current findings suggest a variable rate of cellular aging in the brain and a localized significant increase in cellular aging in the HIPP of MDD subjects, concordant with previous findings pointing to a reduction of hippocampal volume in MDD^34^ and in animal models of stress.^35^

Telomere length is negatively correlated with age especially in dividing cells such as blood.^16^ In this study, however, no strong correlation was observed between age and telomere length in any of the brain regions surveyed (Supplementary Figure 1). This lack of correlation is consistent with other reports using post-mortem brain samples and investigating both gray and white matter.^9, 17, 32^ Thus, telomere shortening with age is probably a phenomenon mainly associated with tissues containing a high ratio of dividing vs non-dividing cells which is not the case in brain tissue. Interestingly, differences in telomere length have been also observed within the same individual. In a recent study by Holstege *et al.*,^36^ telomere length was quantified in 15 tissues, including occipital cortex and whole blood, from a 115-year-old female. They observed that peripheral blood cells have the shortest telomeres while the occipital cortex has the longest, confirming the differential effect of aging on dividing vs non-dividing tissue. We observed variable telomere length also within the brain, with the DLPFC having the shortest telomeres and the SN followed by the HIPP having the longest telomeres regardless of the diagnosis (Figure 1). These results suggest that telomere shortening occurs as a consequence of cells dividing, but also suggests that other factors within the brain can contribute to the maintenance of telomere length.

Previous studies have shown that MDD diagnosis, early life adversity, maternal stress during pregnancy^37^ and care-giver stress all lead to significant decreases in peripheral telomere length and advanced cellular aging, for a comprehensive review see the study by Wolkowitz *et al.*^4^ Our observation of decreased telomere length in the HIPP of MDDs is of interest because (1) the HIPP, a primary limbic region, is associated with the pathophysiology of depressive disorders;^38^ and (2) there are reports of volumetric hippocampal decreases in MDDs compared with controls, and (3) the HIPP is a site of significant neurogenesis in the adult brain.^39^ The decrease in hippocampal volume in MDD may be correlated with shortening of telomeres in this brain region. It is noteworthy that shorter leukocytes telomeres have been associated with smaller hippocampal volume in imaging studies.^40^ To investigate the relationship between telomere maintenance/or stress response genes have on telomere length between the regions with the largest differences, we compared gene expression in DLPFC (shortest average telomere length) vs SN (longest telomere length) in control subjects to exclude any diagnosis or medication effect. We observed an overall significant decrease in telomere maintenance gene expression in the DLPFC compared with the SN. Most genes forming the telomerase complex (TERC, GAR1, DKC1, NHP2 and NOP10) and the shelterin complex (TPP1, TERF1, TERF2IP, TINF2 and POT1) were expressed at higher levels in the SN when compared with the DLPFC, possibly indicating that telomeres are better maintained in the SN. We cannot, however, exclude an effect of the ratio of dividing (glial cells) versus non-dividing cells (neurons) on observed differences in telomere length between the two regions. Other studies of telomere length of glial cells and neurons could specifically investigate that possibility.

We also compared expression of genes involved in stress response and telomere maintenance in the HIPP of MDDs and controls using a NanoString custom gene expression assay. The GPR37 and HSPA2 were found to have significantly decreased gene expression in MDDs compared with controls (Figure 3). The GPR37 gene is involved in cyclic AMP signaling and in neuroprotection,^41^ while HSPA2 is a chaperone and was previously shown to have significantly decreased gene expression in the DLPFC and anterior cingulate cortex of MDDs, in a different cohort, compared with controls.^42, 43^ Meanwhile, two nuclear receptor transcription factors (PPARD and PPARG) involved in neuroprotection, energy homeostasis, inflammation and response to oxidative stress^44, 45^ were expressed at higher levels in the HIPP of MDD subjects (Figure 3).

Finally, key players in regulation of the response to stress showed significantly altered gene expression in the HIPP of MDDs. FKBP5 was upregulated, while HSPA2 and CRH were downregulated in MDD subjects (Figure 3). Increased levels of FKBP5 gene expression have been previously demonstrated in the HIPP of rodents exposed to chronic mild stress^46^ and following treatment with dexamethasone.^47, 48^ Upregulation of FKBP5 gene expression is controlled by glucocorticoids ^49^ and has also been associated to a single nucleotide polymorphism within its intron 2, which lies within a hormone response element sensitive to glucocorticoids,^50, 51^ both of which could explain the increased levels observed in the present study. FKBP5 along with co-chaperones HSP90 and HSPA2 (also known as hsp70) are part of a short negative feedback loop, which regulates the glucocorticoid receptor (GR).^47^ Increased expression of FKBP5 has been demonstrated to retain the GR within the cytosol thus, impeding its translocation to the nucleus and subsequent activation of stress response GR-target genes.^47^ One of these target genes is CRH, which is considered to be a dominant regulator of the body's physiological reaction to stressors through CRH release from the hypothalamus during times of stress,^52, 53^ and was found to be downregulated in MDDs in this study. The observed gene expression alterations of FKBP5, HSPA2 and the GR-target gene CRH could explain the susceptibility of the HIPP to the effects of stress and the reduction in telomere length in MDDs observed specifically in this region.

This is to our knowledge the first study to survey telomere length across five brain regions in mood disorders, SZ and controls. However, this study is not without limitations. The variability in telomere length across brain regions and the significant decrease in telomere length in MDD need to be replicated in a larger cohort. The brain is composed of a majority of dividing glial cells with significantly fewer neurons.^54^ Therefore, it is plausible that the observation of decreased telomere length in the HIPP of MDDs is driven by glia, although there is evidence for non-dividing cells, such as neurons, to undergo telomere shortening due to stress.^2^ Thus, since the method employed herein does not distinguish between glial cells and neurons further investigation of telomere length using cell-specific fractions is required. The results also connect alterations of gene expression of stress response genes in MDD and telomere shortening in the HIPP, known to have sub-regions such as the dentate gyrus able to produce new neurons in adults. More region-specific measurements within the HIPP are required to pinpoint sub-regions and types of cells more susceptible of accelerated cellular aging as measured by telomere length reductions. Finally, it would be of interest to examine this connection further by investigating additional markers of stress, such as cortisol levels and brain-specific isoforms of the GR.

In conclusion, this study shows important differences in telomere length across regions in the brains of psychiatric patients, and presents significant findings that are consistent with an advanced cellular aging in the HIPP of MDDs. This finding leads one to question the interplay between stress, neurogenesis and cellular aging on the pathophysiology of depression.

## Figures and Tables

**Table 1:** Demographic details of the post-mortem sample

	*C*	*BD*	*MDD*	*SZ*	*ANOVA* P-*value*
Average age (±s.d.)	48 (13.0)	52.4 (13.2)	47.3 (11.5)	45.6 (9.0)	0.483
					
*Gender*					0.462
Females	3	5	7	5	
Males	7	5	3	5	
					
pH	6.15 (0.19)	6.52 (0.42)	6.40 (0.39)	6.44 (0.43)	0.244
					
PMI	20.52 (8.25)	22.98 (7.30)	24.76 (7.34)	22.13 (6.38)	0.691

Abbreviations: ANOVA, analysis of variance; BD, bipolar disorder; C, control; MDD, major depressive disorder; PMI; post-mortem interval; SZ, schizophrenia.

**Table 2:** *P*-values and FC comparing telomere length between MDD< BD, SZ and controls across five brain regions, with and without the inclusion of age, pH and PMI as covariates in the model

*Brain region*	*MDD vs C*				*BD vs C*				*SZ vs C*			
	*Uncorrected*		*Corrected*		*Uncorrected*		*Corrected*		*Uncorrected*		*Corrected*	
	P-*value*	*FC*	P-*value*	*FC*	P-*value*	*FC*	P-*value*	*FC*	P-*value*	*FC*	P-*value*	*FC*
Amygdala	0.62	1.08	0.37	1.20	0.21	1.24	0.06	1.42	**0.05**	1.31	**0.05**	1.44
DLPFC	0.41	−1.25	0.42	−1.22	0.42	−1.24	0.65	−1.12	0.27	−1.35	0.20	−1.39
Hippocampus	**0.0048**	−1.81	**0.02**	−1.87	0.09	−1.47	0.19	−1.34	0.10	−1.51	0.10	−1.46
Nucleus accumbens	0.57	−1.08	0.78	−1.05	0.46	1.10	0.48	1.11	0.27	1.17	0.23	1.19
Substantia nigra	0.48	1.22	0.06	1.62	0.44	−1.28	0.68	1.13	0.60	−1.15	0.82	1.07

Abbreviations: BD, bipolar disorder; C, control; DLPFC, dorsolateral prefrontal cortex; FC, fold change; MDD, major depressive disorder; PMI, post mortem interval; SZ, schizophrenia.

*P-values in bold are significant at ⩽0.05.
