# Supplementary material for: Variable telomere length across post-mortem human brain regions and specific reduction in the hippocampus of major depressive disorder
Source: Transl Psychiatry. 2015 Sep 15;5(9):e636–. doi: 10.1038/tp.2015.134 (PMC5068804; doi:10.1038/tp.2015.134)

Supplementary Information for:

Variable telomere length across post-mortem human brain regions and specific reduction in the hippocampus of major depressive disorder

Firoza Mamdani[1], Brandi Rollins[1], Ling Morgan[1], Richard M. Myers[2], Jack D. Barchas[3], Alan F. Schatzberg[4], Stanley J. Watson[5], Huda Akil[5], Steven G. Potkin[6], William E. Bunney[6], Marquis P. Vawter[1], Adolfo Sequeira[1]*

[1] Functional Genomics Laboratory, Department of Psychiatry and Human Behavior, University of California, Irvine
[2] HudsonAlpha Institute for Biotechnology, Huntsville, AL, USA
[3] Department of Psychiatry, Weill Cornell Medical College, New York, NY, USA
[4] Department of Psychiatry & Behavioral Sciences, Stanford University, Palo Alto, CA, USA
[5] Molecular and Behavioral Neurosciences Institute, University of Michigan, Ann Arbor, MI, USA
[6] Department of Psychiatry and Human Behavior, University of California, Irvine

Supplementary Table 1: Genes surveyed for gene expression using NanoString, including their accession number, target region and the NanoString probe sequence.

Gene Symbol	Gene Name	Accession	Target Region	Target Sequence
CRH	corticotropin releasing hormone	NM_000756.1	681-781	ACCTCCTCCGGGAAGTCTTGGAAATGGCCAGGGCCGAGCAGTTAGCACAGCAAGCTCACAGCAACAGGAAACTCATGGAGATTATTGGGAAATAAAACGG
DKC1	Dyskerin	NM_001363.3	2255-2355	GATGGGCCTGGTAGTTTTCCATCTTGTTCTGGCCTAGAGGTCAGTCCTTTGCACTTCCTCAAAGCTTGTGTACAGTGCTCACCTAAATCCATCTGACTAC
FKBP5	FK506 binding protein 5	NM_001145775.1	540-640	ACCATTTGTCTTTAGTCTTGGCAAAGGCCAAGTCATCAAGGCATGGGACATTGGGGTGGCTACCATGAAGAAAGGAGAGATATGCCATTTACTGTGCAAA
GAR1	Nucleolar protein family A, Member 1 (NOLA1)	NM_032993.2	641-741	AGGTGGAGGTGGGGGCTTCAGAGGAGGAAGAGGTGGTGGTTTCAGAGGGAGAGGACATTAAGTGAAACAGTTGACAGACATCACCAGTTGACTTCTGCAT
GPR37	G-protein coupled receptor 37	NM_005302.2	1890-1990	GCTCCGGCAGAAAGGTGCATTATTAAGATCTCTCCTGATTTACCAGACACCATCTATGTTCTAGCCCTCACCTACGACAGTGCGAGACTGTGGTGGTATT
HSPA2	heatshock 70kDa protein 2	NM_021979.3	2095-2195	AAGATCCTCGACAAGTGTCAGGAGGTGATCAACTGGCTCGACCGAAACCAGATGGCAGAGAAAGATGAGTATGAACACAAGCAGAAAGAGCTCGAAAGAG
NHP2	Nucleolar protein family A, Member 2 (NOLA2)	NM_017838.3	315-415	AGCAGATTCGGCGCGGGGTGAAAGAGGTTCAGAAATTTGTCAACAAAGGAGAAAAAGGGATCATGGTTTTGGCAGGAGACACACTGCCCATTGAGGTATA
NOP10	Nucleolar protein family A, Member 3 (NOLA3)	NM_018648.3	326-426	GGAGACTCCGTAACCAAACTCTTCGGACTGTGAGCCCTGATGCCTTTTTGCCAGCCATACTCTTTGGCATCCAGTCTCTCGTGGCGATTGATTATGCTTG
NPY	neuropeptide Y	NM_000905.2	270-370	AGAGATATGGAAAACGATCCAGCCCAGAGACACTGATTTCAGACCTCTTGATGAGAGAAAGCACAGAAAATGTTCCCAGAACTCGGCTTGAAGACCCTGC
NR3C1	nuclear receptor subfamily 3, group C, member 1	NM_001018077.1	1665-1765	GCTTTCTCCTCTGGCGGGAGAAGACGATTCATTCCTTTTGGAAGGAAACTCGAATGAGGACTGCAAGCCTCTCATTTTACCGGACACTAAACCCAAAATT
NR3C2	nuclear receptor subfamily 3, group C, member 2	NM_000901.3	430-530	CCTACAGAGAGGACCGATGAGAATAACTACATGGAGATTGTCAACGTAAGCTGTGTTTCCGGTGCTATTCCAAACAACAGTACTCAAGGAAGCAGCAAAG
POT1	protection of telomeres 1 homolog	NM_015450.2	1075-1175	ATGCAGTATTTTGACCTGACTTGTCAGCTCTTGGGCAAAGCAGAAGTGGACGGAGCATCATTTCTTCTAAAGGTATGGGATGGCACCAGGACACCATTTC
PPARA	peroxisome proliferator – activated receptor, alpha	NM_001001928.2	5220-5320	GGGTGTGTTTGCTATACGAACATAATGGACGTGAAGTGGGGCAGAAACCCAGAACTCAGCATTCAAGGATGCCCAGGAGAGCTGTCCCTGTTTTAAAGAG
PPARD	peroxisome proliferator – activated receptor, delta	NM_006238.4	107-207	GTAGGCAGCCGGGACAGTGTTGTACAGTGTTTTGGGCATGCACGTGATACTCACACAGTGGCTTCTGCTCACCAACAGATGAAGACAGATGCACCAACGA
PPARG	peroxisome proliferator – activated receptor, gamma	NM_015869.3	1035-1135	GAGCAAAGAGGTGGCCATCCGCATCTTTCAGGGCTGCCAGTTTCGCTCCGTGGAGGCTGTGCAGGAGATCACAGAGTATGCCAAAAGCATTCCTGGTTTT
RXRA	retinoid X receptor, alpha	NM_002957.4	5050-5150	TACAAATGTAATTTTATCCCTCATGTATACTTGGATATGGCGGGGGGAGGGCTGGGACTGTTTCGTTTCTGCTTCTAGAGATTGAGGTGAAAGCTTCGTC
TERC	telomerase RNA component	NR_001566.1	87-187	CCCGCGCGCTGTTTTTCTCGCTGACTTTCAGCGGGCGGAAAAGCCTCGGCCTGCCGCCTTCCACCGTTCATTCTAGAGCAAACAAAAAATGTCAGCTGCT
TERF1	telomeric repeat binding factor	NM_003218.3	1037-1137	CTGAAAGCAGAATACCTGTTTCAAAGAGTCAGCCGGTAACTCCTGAAAAACATCGAGCTAGAAAAAGACAGGCATGGCTTTGGGAAGAAGACAAGAATTT
TERF2	telomeric repeat binding factor 2	NM_005652.2	2465-2565	TGACATCAACCCTAGACCTGTCTGCCTTGGCATTTGCTGTCAACATCTGCTGGGCTATGTAGGCAGGTTAATCCTCCACTTCTCATGTGGTTGAACCAGT
TERF2IP	telomeric repeat binding factor 2, interacting protein	NM_018975.2	1380-1480	TTGCATTGGAACTGGCACTTATTTTCTGACCATCGCTGCTGTTGCTCTGTGAGTCCTAGATTTTTGTAGCCAAGCAGAGTTGTAGAGGGGGATAAAAAGA
TERT	telomerase reverse transcriptase	NM_198255.2	2137-2237	GTGCTGCGTGTGCGGGCCCAGGACCCGCCGCCTGAGCTGTACTTTGTCAAGGACAGGCTCACGGAGGTCATCGCCAGCATCATCAAACCCCAGAACACGT
TINF2	TERF1 (TRF1)-interacting nuclear factor 2	NM_001099274.1	1684-1784	CTAGACCTTTGTGATAGAACTAAAATGCTCTCTGTACTCTAGTCTCCTGCCTCCTCAGCTCTGCAAGTAGTTTAGTAGGAATGAAGTGGAAGTCCAGGCT

<u>Supplementary Table 2:</u> Effect of covariates on telomere length within individual brain regions,p- values are shown.

Brain Region	Age	Gender	pH	PMI
**Amygdala**	0.655	0.290	0.137	0.593
**DLPFC**	0.079	0.187	0.470	0.711
**Hippocampus**	0.268	0.130	0.168	0.343
**Nucleus Accumbens**	0.645	0.232	0.757	0.261
**Substantia Nigra**	0.222	0.206	0.002	0.705

Supplementary Figure 1: Correlation between age and telomere length across brain regions. (A) Table of Pearson correlation (r) and P-values for the correlation between age and telomere length across brain regions. (B) Graphical representation of correlation between age (on the X-axis) and telomere length (on the Y- axis) for each brain region.

A

Brain Region	r	P-value
**Amygdala**	-0.147	0.364
**DLPFC**	-0.264	0.109
**Hippocampus**	-0.127	0.435
**Nucleus Accumbens**	0.004	0.980
**Substantia Nigra**	-0.259	0.112

B

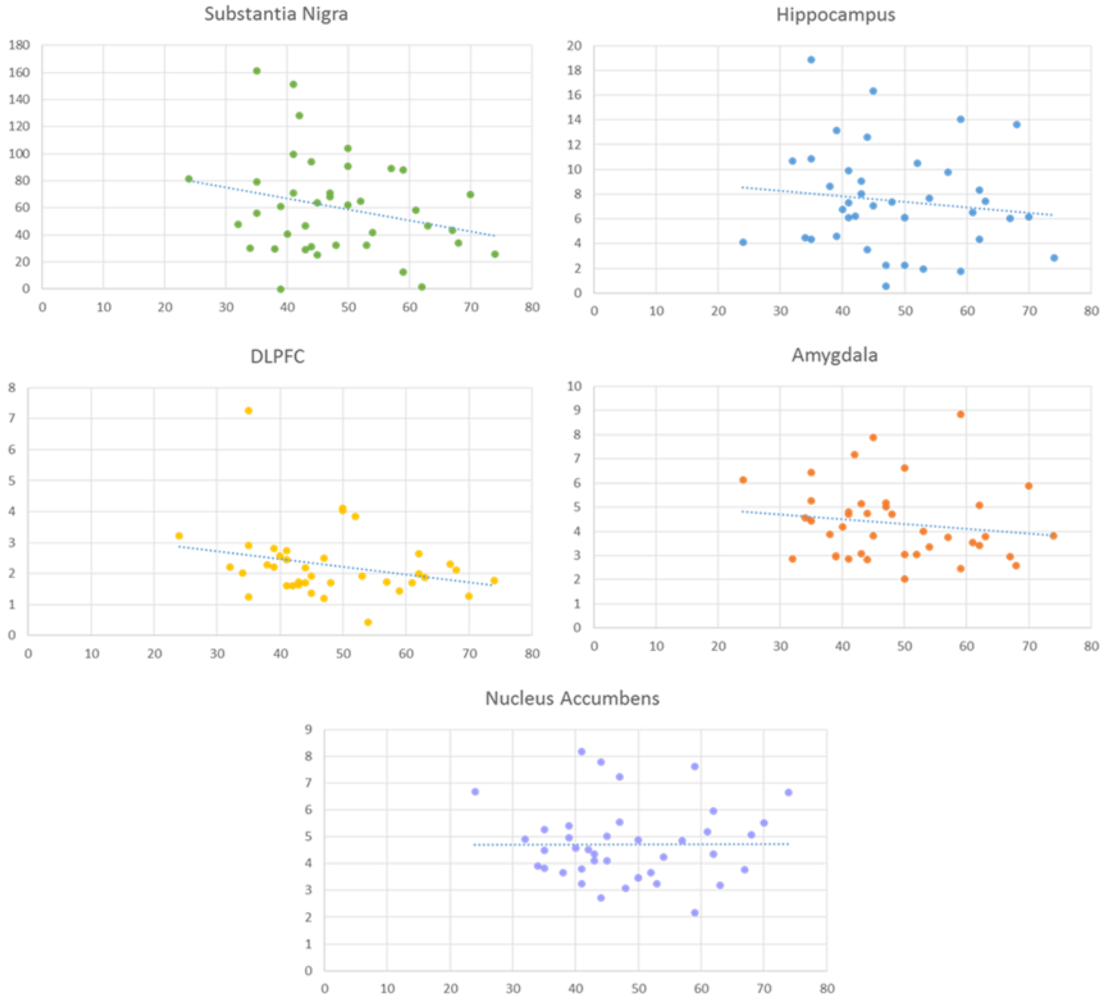

Supplementary Figure 2: Telomere length distribution in controls and MDD in the Hippocampus. MDDs were found to have significantly reduced telomere length compared to controls (P=0.004) in the hippocampus, following correction for the effect of age.

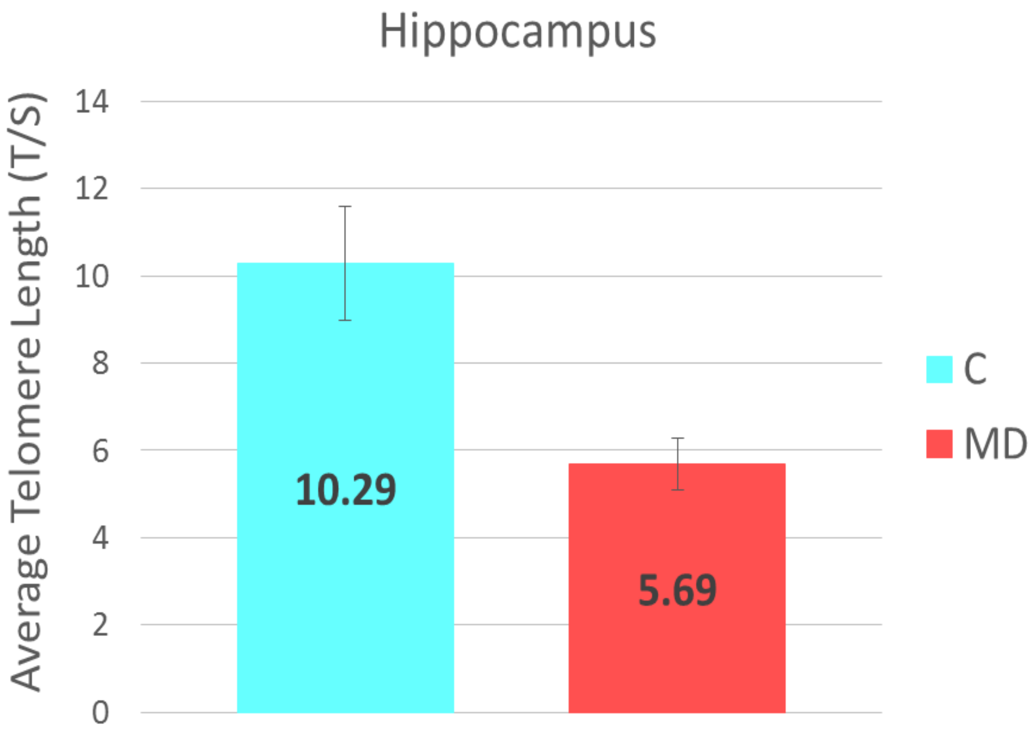

Supplement: Supplementary Information [file tp2015134x1.pdf]
